# 心肺运动试验在肺癌手术风险评估中的应用

**DOI:** 10.3779/j.issn.1009-3419.2011.07.09

**Published:** 2011-07-20

**Authors:** 春婷 谭, 浩彦 王, 永 崔, 志 高

**Affiliations:** 1 100050 北京，首都医科大学附属北京友谊医院呼吸内科 Department of Respiratory Medicine, Beijing Friendship Hospital, Affiliated to Capital University of Medical Sciences, Beijing 100050, China; 2 100050 北京，首都医科大学附属北京友谊医院胸外科 Department of Thoracic Surgery, Beijing Friendship Hospital, Affiliated to Capital University of Medical Sciences, Beijing 100050, China

**Keywords:** 心肺运动试验, 肺肿瘤, 肺切除术, 最大摄氧量, 无氧阈, Cardiopulmonary exercise testing, Lung neoplasms, Lung resection, Maximal oxygen uptake, Anaerobic threshold

## Abstract

心肺运动试验已经成为评估肺癌患者手术风险的重要方法，肺癌患者术前进行心肺运动试验，可以对手术风险进行分级，评估适应证，以降低患者术后并发症的发生率和死亡率。

## Abstract (en)

Cardiopulmonary exercise testing (CPET) has become an important clinical tool to evaluate exercise capacity and predict outcome for patients with lung cancer being considered for lung resection. CPET can help in stratifying the surgical risk and identify those high-risk patients with lung resection. Preoperative screening using CPET allow the selection of appropriate therapeutic approach to decrease surgical complications and mortality.

肺癌是常见的恶性肿瘤，目前手术仍是早期肺癌有效的治疗方法之一。手术为创伤性治疗，可能引起许多术后并发症甚至导致患者死亡，故术前手术风险评估很有必要^[1]^。目前静息肺功能为评价手术风险的常规方法，但对一些肺功能显示风险增加的患者，需要进一步行心肺运动试验（cardiopulmonary exercise testing, CPET）评估手术风险^[2]^。

## 1 肺癌患者手术风险

对于早期肺癌，尤其是无纵隔淋巴结转移且临床诊断为Ⅰ期-Ⅱ期的非小细胞肺癌（non-small cell lung cancer, NSCLC）患者，手术切除是主要的治疗手段^[3]^。外科手术的目的在于彻底清除病变，对于肺功能良好的患者，手术包括肿瘤的全部切除（解剖上的肺叶切除，双肺叶切除或全肺切除）以及邻近受累结构和纵隔淋巴结清扫，以便准确地分期。对于不能承受广泛肺切除的患者可考虑行局部肺段切除或肺楔形切除术。肺癌手术方法包括开胸手术和电视胸腔镜手术，对大部分患者，为了获得较好的远期效果仍需开胸行标准的根治性手术^[1]^。开胸肺切除手术是创伤性治疗，可引起患者呼吸生理紊乱、肺组织容量减少、膈肌运动障碍等，影响患者的通气功能和换气功能，加之手术操作本身及术前的麻醉、术后伤口疼痛致咳嗽排痰受限、术后血流动力学发生改变、血管床面积缩小、右心负荷加重，都会引起患者呼吸、循环系统紊乱，可导致术后出现并发症，如呼吸衰竭、肺炎、肺不张、急性呼吸窘迫综合征、肺水肿、肺栓塞、心肌梗死、心律失常、心脏衰竭、急性肾功能不全、败血症、休克、死亡等^[4]^。

## 2 肺癌手术风险评估

### 2.1 静息肺功能

开胸手术前一般常规进行静息肺功能检查，常规肺功能指标可反映气道阻塞程度及患者的呼吸储备、呼吸肌强度，可作为预测术后肺部并发症的初筛^[5]^。肺切除手术方式不同，术后并发症发生率也不同，切除肺组织愈多，手术范围越大，术后并发症发生率也越高。美国胸科医师学会（American college of chest physicians, ACCP）建议能耐受肺切除术的肺功能测定值为：①全肺切除：第一秒用力呼气容积（forced expiratory volume in one second, FEV_1_） > 2 L，FEV_1_占预计值> 80%；②肺叶切除：FEV_1_ > 1.5 L（1C）。如果患者存在不能解释的呼吸困难或影像上存在弥漫性肺实质疾病表现，则应测定弥散功能^[2]^。如果FEV_1_或肺一氧化碳弥散量（diffusion capacity for carbon monoxide of the lung, D_L_CO）均 < 80%预计值，应计算预计术后肺功能。如果预计术后FEV_1_ < 40%，或预计术后D_L_CO < 40%，则围手术期死亡率和并发症增加，建议这类患者行CPET^[2, 5]^。

### 2.2 CPET的特点

CPET或运动心肺功能试验是指伴有代谢测定的一项检查，它通过综合心肺功能，在一定功率负荷下检测受试者运动过程中相关生理参数变化（包括运动气体代谢、动态心电图、血压等），评估受试者呼吸、循环、神经、骨骼肌肉等系统整体功能和储备能力，所以它可反映细胞呼吸功能的变化^[6]^。与静态肺功能检查和一般心脏负荷试验不同，它强调运动时心肺功能的相互作用和气体交换作用，综合反映心与肺在一定负荷下通气量、摄氧量和二氧化碳排出量等代谢、通气指标及心电图变化，反映人体的最大有氧代谢能力和心肺储备能力，特别强调心肺功能的同步测定^[7]^。但CPET检查仪器结构较为复杂、检查指标较多、受检者配合程度要求较高、且具有一定的危险性，应严格掌握检查的适应证和禁忌证。尤其对于合并心血管疾病的患者，在检查过程中需密切观察患者反应、血压及心电图情况，而因脑血管疾病或身体畸形不能运动的患者则不能进行该检查^[8]^。

开胸手术可引起全身的系统性炎症反应，氧耗可由静息时的110 mL/(min·m^2^)增加到术后的170 mL/(min·m^2^)，即增加50%。并且，这种高水平的氧耗要持续很长时间，这需要足够的心肺储备才能满足术后氧耗的增加^[9]^。CPET通过增加心肺及氧传递系统的负荷，使受试者的通气量、摄氧量、二氧化碳产生量、脉率及心排血量都相应增加，即增加整个心肺系统及氧运输系统的负荷，在某种程度上与肺切除手术对患者施加的负荷相似，体现了在最大运动负荷下心肺运动耦联的能力，故能比较全面地判断患者对手术的耐受力^[10]^。从而帮助胸外科医师确定手术的适应证，减少术后并发症。早在20世纪70年代就应用于外科手术风险性评估^[11]^。近年来，CPET在肺癌患者手术风险评估领域受到越来越多的关注。

### 2.3 其它肺癌手术风险评估方法

如往返步行试验、6分钟步行试验对预测肺切除手术风险亦有一定价值，多认为往返步行试验中往返小于25次（250 m）或试验过程中血氧饱和度降低 > 4%的患者围手术期死亡率和心肺并发症风险增加，而6分钟步行试验的距离对肺切除术风险的预测作用目前尚无共识^[2, 5]^。登楼试验也可作为预测肺切除患者术后心肺并发症的方法之一，并且相对安全而经济。一般认为登3层楼的患者可行肺叶切除术，登5层楼的患者可耐受全肺切除术^[12, 13]^。登楼试验的层数与最大摄氧量（maximal oxygen uptake, VO_2_max）相关，Beckles等^[14]^认为登楼大于5层的患者VO_2_max/kg > 20 mL/(kg·min)，登楼小于1层的患者VO_2_max/kg < 10 mL/(kg·min)。但相对于CPET而言，登楼试验过程中登楼的速度、每层楼的台阶数、每级台阶的高度、终止运动的指征等尚缺乏统一标准^[2]^。

## 3 CPET在肺癌手术风险评估中的应用

1972年Reichel^[11]^首先报道了CPET在全肺切除手术术前评估的应用，之后CPET在肺切术手术领域的应用受到广泛研究^[15-17]^。迄今，常用评估肺切除手术风险的CPET指标为VO_2_max、最大公斤摄氧量（VO_2_max/kg）、最大摄氧量占预计值%（VO_2_max%）和无氧阈（anaerobic threshold, AT）。

### 3.1 VO_2_max

指细胞最大的摄氧能力，指在运动的最后阶段，即竭尽全力阶段，循环和呼吸系统发挥最大作用时每分钟摄取的氧量，一般等同于最大运动状态下的摄氧量，即峰值摄氧量（VO_2_peak），若考虑到体重的影响，可用最大公斤摄氧量（VO_2_max/kg）表示。VO_2_max取决于循环系统和呼吸系统的功能，故呼吸和循环受限均可导致VO_2_max下降。

1982年Eugene等^[15]^即提出VO_2_max与肺切除术后死亡率密切相关。1984年Smith等^[16]^认为VO_2_max应用体重进行校正，用最大公斤摄氧量（VO_2_max/kg）评估手术风险更科学，提出VO_2_max/kg > 20 mL/(kg·min)手术风险低，VO_2_max/kg < 15 mL/(kg·min)手术风险高。1987年Bechard等^[17]^研究50例肺切除患者，结果认为VO_2_max/kg < 10 mL/(kg·min)为高危患者。BTS在2001年发布的肺癌患者手术指南中认为VO_2_max/kg > 15 mL/(kg·min)的患者手术风险较低，而VO_2_max/kg < 15 mL/(kg·min)的患者手术风险高^[5]^。2007年ACCP指南同样推荐根据VO_2_max/kg值对拟行标准肺切除术的肺癌患者进行危险度分级：VO_2_max/kg在15 mL/(kg·min)-20 mL/(kg·min)的患者一般能耐受手术，死亡率和心肺并发症发生率较低；VO_2_max/kg介于10 mL/(kg·min)-15 mL/(kg·min)，围手术期死亡和心肺并发症发生率增加；VO_2_max/kg < 10 mL/(kg·min)，术后死亡和心肺并发症风险非常高，建议对这类肺癌患者进行非标准手术或采取非手术治疗方法^[2]^。2009年Bobbio等^[18]^入选73例行肺切除术（包括单/双肺叶切除术和肺段切除术）的患者（其中71例为NSCLC），术前进行CPET，观察术后30天手术并发症和死亡率。结果为VO_2_max/kg < 15 mL/(kg·min)的患者肺部并发症的发生率为43%，而VO_2_max/kg > 20 mL/(kg·min)的患者肺部并发症的发生率为9.1%。2009年Brunelli等^[4]^纳入了204例进行肺叶切除术和全肺切除术的患者及59例局限肺切除的患者。术前进行CPET，观察VO_2_max与术后心肺并发症和死亡率的关系。结果表明VO_2_max/kg在预测肺切除患者术后心肺并发症方面是一项独立可信的预测指标，VO_2_max/kg > 20 mL/(kg·min)的患者术后死亡率和心肺并发症发生率低，是可以耐受肺切除术的界限值。VO_2_max/kg < 12 mL/(kg·min)的患者术后死亡率和并发症明显升高，而进行楔形或肺段切除的患者术后并发症的发生率较肺叶或全肺切除术的患者低。研究并建议对于肺癌拟行手术的患者，除目前指南建议之外，应更加广泛的开展术前CPET。

### 3.2 VO_2_max%

最大摄氧量实测值与预计值相比较即VO_2_max占预计值%（VO_2_max%），一般应大于预计值的84%。

1995年Bolliger等^[19]^认为VO_2_max绝对值应根据性别、年龄进行校正，故VO_2_max%比单纯VO_2_max绝对值预测肺切除手术风险更优越。其研究结果认为：VO_2_max% > 75%，手术风险低；VO_2_max% < 60%，手术风险增加；VO_2_max% < 43%，应避免行开胸手术。2000年Brutsche等^[20]^纳入了125例NSCLC行开胸肺切除手术（包括全肺切除、单/双侧肺叶切除、肺段/楔形切除）的患者，术前进行CPET，观察术后30天的并发症和死亡率，结果认为VO_2_max%是预测术后并发症的最佳指标，VO_2_max% > 90%，手术风险低；VO_2_max% < 60%手术风险明显增加。2005年Win等^[1]^认为若将VO_2_max/kg高风险值定为15 mL/(kg·min)则将很多可以从手术中获益的肺癌患者排除在外，比如老年人、身体矮小者和/或女性患者，因为这些人群的VO_2_max/kg正常值就可能低于15 mL/(kg·min)，故认为采用VO_2_max%来判定将更科学。其研究入选101例NSCLC行肺切除术的患者，术前进行静息肺功能及CPET，结果认为VO_2_max% < 50%的肺癌患者的严重并发症（死亡、心肌梗塞、呼吸衰竭）发生率明显高于VO_2_max% > 60%的患者。故认为，VO_2_max% < 50%-60%预计值时，术后死亡率和并发症将增加。2007年Loewen等^[21]^综合了以上的研究方法，入选364例肺癌进行肺切除手术的患者，研究认为VO_2_max/kg < 16 mL/(kg·min)或VO_2_max < 65%的患者术后并发症及死亡率将明显增加。国内李琦等^[22]^亦认为VO_2_max受体重、年龄和性别等影响较大，单纯用绝对值评估手术适应证并不适于所有人群，推荐VO_2_max%作为预测全肺切除术后呼吸衰竭、评估手术适应证的指标。

2007年Benzo等^[23]^对包括955例患者的14项研究进行的一项*meta*分析认为，VO_2_max/kg与VO_2_max%均可对肺切除患者进行危险度分级。

### 3.3 AT

又称乳酸阈或通气阈，机体在负荷递增的运动过程中，有氧代谢不能满足运动肌肉的能量需求，需要通过无氧代谢来增加供能开始时的摄氧量。即尚未发生乳酸性酸中毒时的VO_2_值，反映了机体耐受负荷的潜能。测定方法有乳酸法、V-斜率法（V-Slope）和通气当量法，正常应大于预计VO_2_max的40%以上。相对VO_2_max而言，AT更能反映肌肉线粒体利用氧的能力。AT的测定反映循环系统维持氧运输的能力，且与患者的用力无关。它关系到围手术期ATP有氧代谢内环境的稳定。一些研究者认为对心肺功能不全、静态肺功能认为不宜手术又必须手术的患者，CPET可将围手术期高危患者与低危患者区别开，甚至只需测定AT即可，远不需达到极量运动。Older等^[24]^的研究结果为AT > 11 mL/(kg·min)的患者术后并发症发生率低，而AT < 11 mL/(kg·min)的患者术后并发症明显增加。Agnew^[25]^认为AT至少大于11 mL/(kg·min)方可考虑进行开胸手术。

### 3.4 总结

目前国内外预测肺癌患者进行开胸肺切除术风险预测的指标有VO_2_max/kg、VO_2_max%和AT。一般认为VO_2_max/kg≥20 mL/(kg·min)或VO_2_max%≥75%，可行肺切除术；15 mL/(kg·min)≤VO_2_max/kg < 20 mL/(kg·min)或60%≤VO_2_max% < 75%，手术风险低；10 mL/(kg·min)≤VO_2_max/kg < 15 mL/(kg·min)或43%≤VO_2_max% < 60%，手术风险高；VO_2_max/kg < 10 mL/(kg·min)或VO_2_max% < 43%，应禁忌开胸手术。AT≥11 mL/(kg·min)手术风险低，AT < 11 mL/(kg·min)手术风险高。

综上所述，CPET是一项客观评价心、肺功能的无创检查技术，能比较全面地判断患者对手术的耐受力，建议老年或有心、肺等合并症及静态肺功能显示手术临界高风险的肺癌患者，术前行CPET进一步评估，以指导临床，从而降低肺癌患者手术并发症的发生率和死亡率。
